# Investigating the influence of contributing factors and predicting visibility at road link-level

**DOI:** 10.1016/j.heliyon.2019.e02105

**Published:** 2019-07-23

**Authors:** Srinivas S. Pulugurtha, Ajinkya S. Mane, Venkata R. Duddu, Christopher M. Godfrey

**Affiliations:** a The University of North Carolina at Charlotte, 9201 University City Boulevard, Charlotte, NC 28223-0001, USA; b The University of North Carolina at Asheville, 1 University Heights, CPO # 2450, Asheville, NC 28804-8511, USA

**Keywords:** Environmental sciences, Elevation, Weather station, Regression, Visibility, HRRR, Precipitation, Sensor

## Abstract

Data from weather stations at airports, far away locations or predictions using macro-level data may not be accurate enough to disseminate visibility related information to motorists in advance. Therefore, the objective of this research is to investigate the influence of contributing factors and develop visibility prediction models, at road link-level, by considering data from weather stations located within 1.6 km of state routes, US routes and interstates in the state of North Carolina (NC). Four years of meteorological data, from January 2011 to December 2014, were collected within NC. Ordinary least squares (OLS) and weighted least squares (WLS) regression models were developed for different visibility and elevation ranges. The results indicate that elevation and cloud cover are negatively associated with low visibility. The chances of low visibility are higher between six to twelve hours after rainfall when compared to the first six hours after rainfall. A visibility sensor was installed at four different locations in NC to compare hourly visibility from the selected regression model, High-Resolution Rapid Refresh (HRRR) data, and the nearest weather station. The results indicate that the number of samples with zero error range was higher for the selected regression model compared with the HRRR and weather station observations.

## 1 Introduction

From the year 2007 to the year 2016, on average, 1,235,145 weather-related crashes were reported in the United States. Each year, on average, 5,376 people are killed and over 418,000 people are injured in weather-related road crashes (U.S. Department of Transportation, 2018). Wet pavement, rain, sleet or snow, and fog are top reasons for weather-related crashes. In terms of fog-related crashes, in the state of North Carolina (NC), 19,188 crashes were reported from the year 2003 to the year 2012 (Oliver, 2013). Out of these fog-related crashes, 43% of the crashes occurred between the months of November to January, and 49% of the crashes occurred during early morning hours from 5:00 AM to 7:00 AM.

Fog and low visibility are localized phenomena. However, it is expensive to install visibility sensors every few kilometers along a road. Therefore, predicting fog events, disseminating that information to motorists beforehand, and exploring technologies to dynamically adjust speed limits is vital to avoid fog-related crashes.

Past researchers have adopted statistical or numerical modeling techniques to predict fog events. Vislocky and Fritsch (1997) considered predictor variables such as wind speed, wind direction, sea level pressure, dewpoint, and dewpoint depression (the difference between air temperature and dew point temperature), precipitation opaque cloud amount, and cloud cover to predict visibility. Likewise, Tardif and Rasmussen (2008) stated that most of the fog events occurred at high elevation due to upslope flow and attendant lowering of the cloud base. Hilliker and Fritsch (1999) investigated logistic regression to predict visibility. Likewise, Bocchieri et al. (1974) and Hanesiak and Wang (2005) studied low visibility issues using a statistical approach. Marzban et al. (2007) compared the neural network model with linear and logistic models to predict fog events. They concluded that the neural network performs better compared to logistic or linear models. Bergot et al. (2005), Gultepe et al. (2006), Bott et al. (1990), and Bott (1991) explored numerical modeling techniques to predict visibility. Gultepe et al. (2007) conducted a detailed literature review related to visibility prediction models and issues.

Motorists are often caught unaware by sudden reductions in visibility (Hamilton et al., 2014). Low visibility conditions can result in severe injuries, as some motorists choose much lower speeds than other motorists. Multiple vehicles (i.e., more than two) may collide in these conditions, often resulting in rear-end road crashes. Kang et al. (2008) examined the effect of reduced visibility due to fog on car-following performance and concluded that motorists face difficulty while following the lead vehicle in foggy conditions. Abdel-Aty et al. (2011) concluded that lighting conditions have a catalytic effect in fog-and smoke-related crashes.

Ahmed et al. (2014) examined the use of meteorological data collected at airports to assess road crashes at locations with recurrent fog problems. Typically, automated sensors at airport locations collect at least hourly observations and those observations are available in near real-time. The meteorological observations reported at eight airports in Florida were paired with crash data in fog-prone counties, to show that reduction in visibility is statistically related to nearby crash occurrences. It was concluded that real-time meteorological data from airports can be used for nearby roads (i.e., within 8 km) to mitigate the increased risk of limited visibility.

Applying fog prediction models to predict low visibility events could help to inform motorists in advance. Additionally, accurate meteorological data are essential for collision analyses to establish contributing factors associated with these collisions. However, crash investigations at many locations rely on observational data from distant weather stations that may have recorded very different meteorological conditions. It is possible to improve the reliability of predicted low visibility conditions through models developed using nearby weather station data or via technologies for reducing crashes or identifying weather-related contributing factors in collision analyses.

Past studies have investigated the contribution of several meteorological predictors on visibility. However, the influence of rainfall in past hours, the time-of-the-day, and the presence of water bodies within the vicinity were not explored in the past. Also, the influence of meteorological factors on visibility could vary with the elevation and was not explored in the past. Considering all visibility records may reduce the applicability of models for road link-level prediction. These factors were accounted for by modeling low visibility conditions in this research.

Numerical weather prediction models such as the North American Regional Reanalysis (NARR) and High-Resolution Rapid Refresh (HRRR) forecasts can help to analyze weather conditions to some extent. However, the applicability of these models at road link-level is unclear. Using low visibility meteorological data at weather stations near traffic links may help improve the accuracy of the visibility prediction models and assist with the dissemination of such information to motorists or dynamically adjust speed limits in a timely manner. Therefore, the objectives of this research study are:

1. to investigate the influence of contributing factors,
2. to develop visibility prediction models for different visibility and elevation ranges, and,
3. to compare visibility from different data sources.

This research focuses on considering data from only weather stations located within 1.6 km of highways and developing regression models to predict visibility. Collecting meteorological data within the vicinity of road links and then developing statistical models will help to identify the factors influencing low visibility conditions at road link-level in addition to improving the accuracy of the predictions.

## 2 Methodology

The methodology adopted in this research includes the following steps.

1. Select weather stations close to the road network and collect meteorological data
2. Process data
3. Develop regression models to predict visibility
4. Compare visibility from the developed model and other data sources

Each step is explained next in detail.

### 2.1 Select weather stations close to the road network and collect meteorological data

The Integrated Surface Database (ISD) comprises hourly meteorological data from over 35,000 locations worldwide. This dataset is maintained by the National Oceanic and Atmospheric Administration (NOAA)/National Centers for Environmental Information (NCEI) (Del Greco et al., 2006; Smith et al., 2011). The database includes visibility, 2-m air temperature, dewpoint temperature, wind speed, atmospheric pressure, precipitation, and current weather conditions. Some stations also collect snow depth and snowfall information.

Typically, the source of the meteorological dataset is weather stations located at airports. Before distribution/sharing, the ISD database undergoes a meticulous quality control process (Smith et al., 2011; Lott, 2004). In spite of rigorous quality control, there are data quality issues with the database (Godfrey, 2015). Additional quality assurance algorithms are therefore applied at the regional-level.

**Fig. 1:**
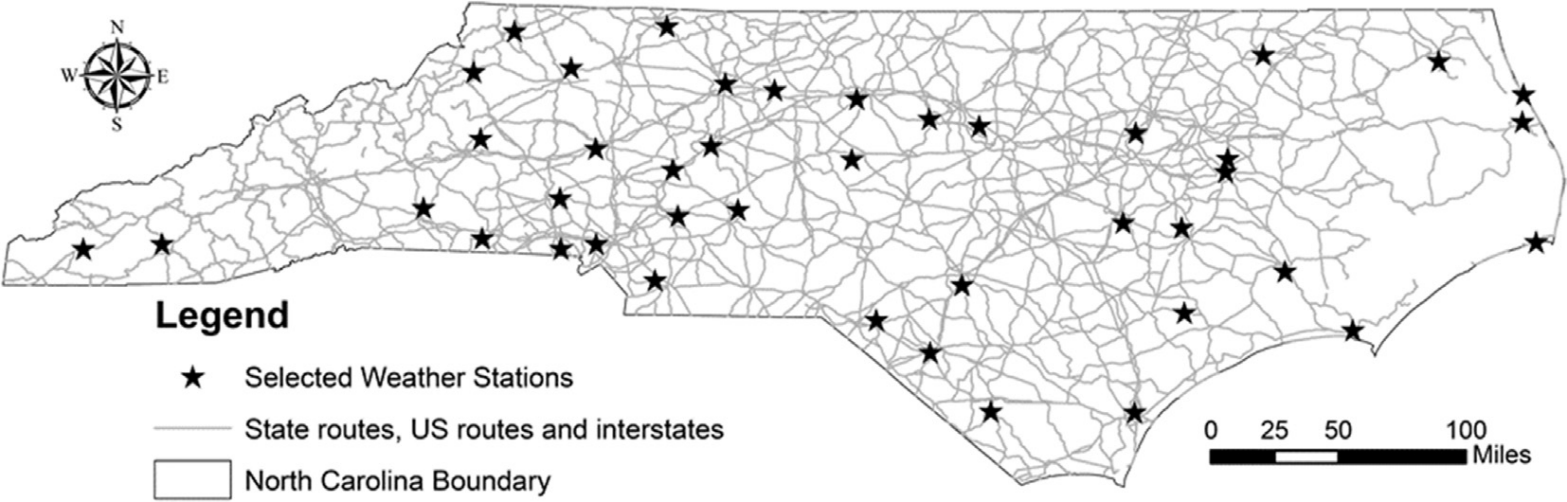
Selected weather stations for model development.

For predicting fog events for motorists, 42 ISD locations were selected in NC. These locations are within 1.6 km from state routes, US routes, and interstates (Fig. 1). Four years of meteorological data, from January 2011 to December 2014, were collected and processed for modeling to predict visibility and validate the results.

### 2.2 Process data

In this step, the missing values were deleted from the database. The elevation (m), cloud cover (%), wind speed at 10-m above ground level (AGL) (m/s), and amount of precipitation (mm) were considered as the predictor variables.

Oliver (2013) stated that fog-related crashes were more likely to occur during the morning hours (5 AM to 7 AM). A significant number of these fog-related crashes during the morning hours are due to low visibility conditions (49%). In addition, the literature review indicated that precipitation is a governing factor in low visibility conditions. Therefore, the precipitation in the previous hours and time-of-the-day could influence the formation of fog. Therefore, binary variables such as the occurrence of rainfall in the past three hours (Rain0-3), three to six hours (Rain3-6), six to twelve hours (Rain6-12), and twelve to twenty-four hours (Rain12-24) were generated and added to the database. Also, six binary variables were added related to the time-of-the-day variable: 12 AM to 4 AM (0am-4am), 4 AM to 8 AM (4am-8am), 8 AM to 12 PM (8am-12pm), 12 PM to 4 PM (12pm-4pm), 4 PM to 8 PM (4pm-8pm), and 8 PM to 12 AM (8pm-12am).

According to NOAA, fog consists of a collection of suspended water droplets or ice crystals near Earth's surface that leads to a reduction of horizontal visibility below 1 km (Glickman, 2000). Therefore, water bodies within the vicinity of a weather station could influence the formation of fog at the weather station. In this research, the presence of water bodies within 1.6 km radius of each selected weather station was captured using ArcGIS and was represented as a dichotomous variable.

Dew point depression (‘tair_dew’ in Kelvin) was computed as a continuous variable by computing the difference between air temperature and dew point temperature for every hour.

### 2.3 Develop regression models to predict visibility

Elevation plays an important role in low visibility events. Further, only 9% of recorded visibility values at the selected 42 ISD locations are less than 2,000 m. Considering all recorded visibility values for modeling may result in skewed/biased predictions. Therefore, linear regression models were developed to predict visibility using data for different visibility and elevation ranges.

Per the International standard, visibility range is classified into eight different classes (Cho and Kim, 2005). They are:

1. Dense fog <40 m
2. Thick fog 40 m to 200 m
3. Fog 200 m to 1,000 m
4. Mist/haze 1,000 m to 2,000 m
5. Poor visibility 2,000 m to 4,000 m
6. Moderate visibility 4,000 m to 10,000 m
7. Good visibility 10,000 m to 40,000 m
8. Excellent visibility >40,000 m

In this research, both fog and non-fog events were considered to eliminate bias and investigate the influence of contributing factors. To ensure that the sample is adequate and to attain statistically meaningful results, the visibility range was classified into four groups: less than 15,000 m, less than 10,000 m, less than 5,000 m, and less than 2,000 m. Visibility less than 15,000 m includes visibility values ranging from dense fog to good visibility while visibility less than 10,000 m includes visibility values ranging from dense fog to moderate visibility. Visibility less than 5,000 m includes visibility values ranging from dense fog to poor visibility and visibility less than 2,000 m includes visibility values ranging from dense fog to mist/haze conditions. Further, for each visibility range, elevation was further classified into five groups: less than 50 m, 50 m to 250 m, 250 m to 750 m, and greater than 750 m.

Ordinary least squares (OLS) and weighted least squares (WLS) regression models were developed using STATA to examine the influence of predictor variables on visibility. OLS regression is the simplest form of regression and tries to minimize the residual sum of squares (RSS). In OLS regression, each observation receives equal weight (irrespective of the distance between the observation and population mean) so as to minimize the RSS. Contrarily, weights are used to minimize the RSS in the case of WLS regression. The observations closer to the population mean receive more weight. For example, *weight*_*i*_ = 1/variance_i_ (Gujarati et al., 2012), where “i” is the individual observation.

Based on the visibility and elevation ranges, data were used for the development of regression models. The predictor variables such as elevation, cloud cover, wind speed at 10-m, dewpoint depression, the time-of-the-day, rainfall in past hours, and the presence of water bodies within 1.6 km of the weather stations were considered for the model development. All the predictor variables were initially considered in the model development and the predictor variables with p-value greater than 0.05 were removed one at a time (predictor variable with highest p-value was removed first followed by redeveloping the regression model). This method is also known as the backward elimination method. The visibility value is considered as the dependent variable.

Altogether, twenty OLS and twenty WLS regression models were developed in this research. Predictor variables were identified as a significant variable if the p-value is less than 0.05 (at a 95% confidence interval). The performance of the developed models was assessed using statistical measures such as R-square, adjusted R-square, Akaike information criterion (AIC), and root mean square error (RMSE) in addition to the sign of the computed coefficient for each significant predictor variable. The best-fitted regression model was selected based on the statistical measures and their applicability for predicting visibility at link-level.

### 2.4 Compare visibility from the developed model and other data sources

A visibility sensor was installed at four different locations across NC. Table 1 summarizes the installation locations and dates of installation. Hourly visibility values observed at each visibility sensor location are considered as the actual visibility around the installed location. Typically, meteorological data are captured hourly at weather stations located at the airports. Therefore, with respect to each visibility sensor location, the nearest weather station data were acquired to compare with the visibility values from both data sources.

**Table 1:** Weather monitoring stations and HRRR grid point from visibility sensor.

Visibility Sensor	Date Installed	Date Uninstalled	# Days	Weather Monitoring Station	Distance between Sensor and Weather Station (Km)	Distance between Sensor and Nearest HRRR Grid Point (Km)
UNC Charlotte	12/15/2016	2/2/2017	47	Charlotte/Douglas International Airport (CLT)	21.17	1.68
Shelby	2/2/2017	2/23/2017	21	Lincolnton–Lincoln County Regional Airport	6.82	0.63
Lenoir	2/23/2017	3/23/2017	27	Boone Airport	10.98	1.45
UNC Asheville	3/23/2017	4/13/2017	21	Asheville Regional Airport	21.14	0.65

In addition, visibility values from the HRRR model were compared with the visibility values from the visibility sensor. HRRR is a NOAA real-time 3 km resolution model that assimilates radar data. Along with visibility, the model forecasts meteorological parameters such as 2-m air temperature, 2-m dewpoint temperature, 2-m relative humidity, 10-m zonal and meridional wind speeds, accumulated precipitation, total cloud cover, surface ceiling height, skin temperature, moisture availability, and downward shortwave radiation.

To check the predictability of the best-fitted regression model, the meteorological data (predictor variables) such as 2-m air temperature, 2-m dew point temperature, cloud cover, wind speed at 10-m AGL, precipitation, and elevation were collected from the nearest weather stations (because the installed visibility sensor captures only visibility conditions) and added to the developed regression model. The estimated visibility from the regression model was also compared with the visibility sensor.

The nearest HRRR grid point and the nearest weather station from the visibility sensor are presented in Table 1. Only visibility values less than 2,000 m (ranging from dense fog to mist/haze) were selected from the visibility sensor and were compared with the nearest HRRR grid point, the nearest weather station, and the best-fitted regression model (same date and hour).

For comparison purposes, the hourly visibility from each data source was classified as per the international standards (Cho and Kim, 2005).

1. Dense fog (visibility less than 40 m) represented as 1
2. Thick fog (visibility between 40 m to 200 m) represented as 2
3. Fog (visibility between 200 m to 1,000 m) represented as 3
4. Mist/haze (visibility between 1,000 m to 2,000 m) represented as 4

The errors were computed by computing the difference between the visibility categories (Eq. 1).

(1) $$\text{Error}\;=\text{Visibility}\;\text{Classification}\;{\;}_{Visibility\;sensor}-\;{\text{Visibility}\;\text{Classification}\;}_{HRRR,\;\;\;Weather\;Station,\;\;\;Regression\;Model}$$

## 3 Results

**Fig. 2:**
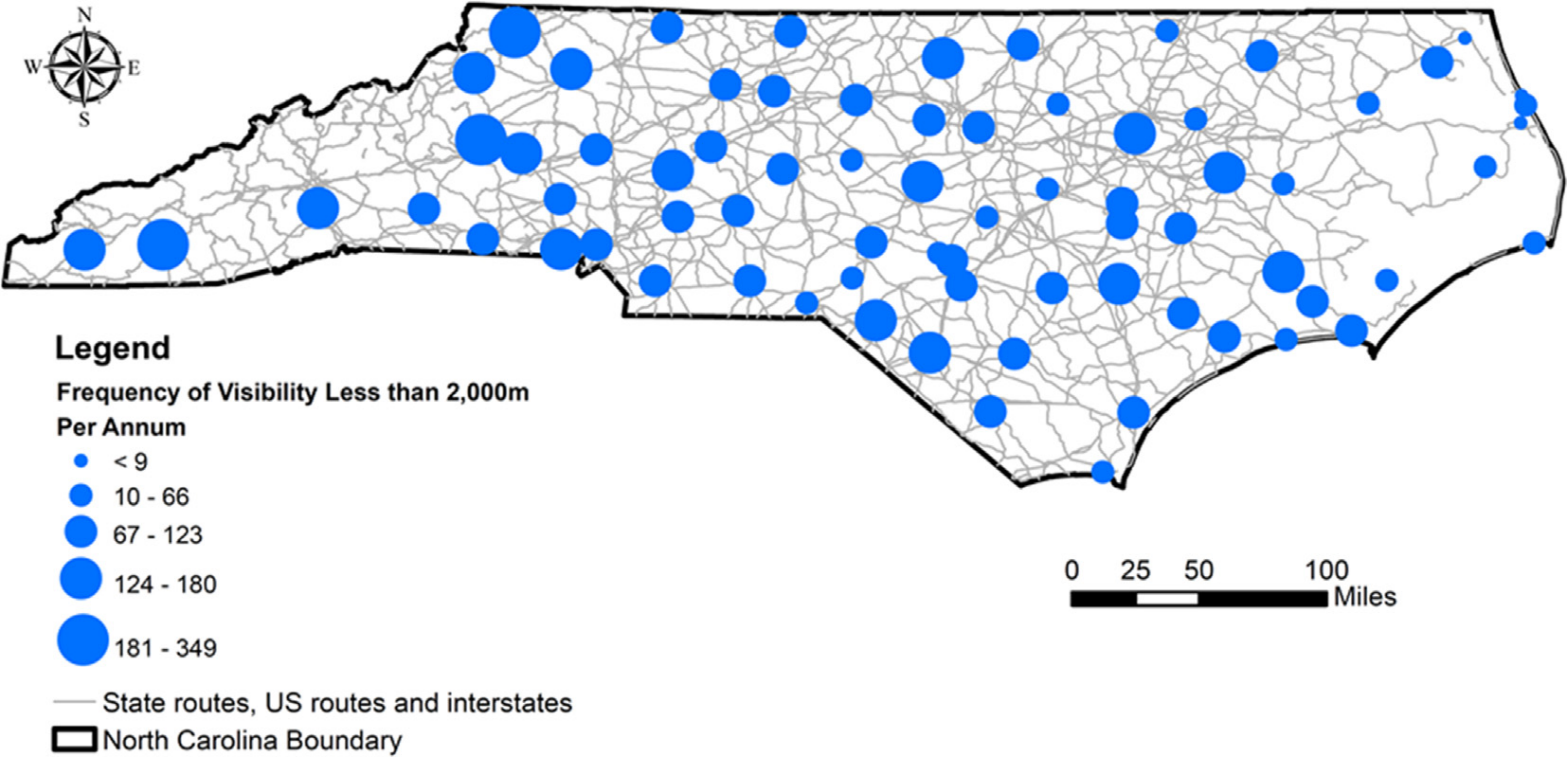
Average annual frequency of visibility less than 2,000 m in North Carolina.

Over the span of 11 years, from the year 2005 to the year 2014, the average annual frequency of visibility less than 2,000 m was reported at 73 weather stations in NC (Fig. 2). The top five locations in NC were Macon County, Ashe County, Asheville, Nash County (Rocky Mount), and Boone. The frequency of low visibility conditions was observed to be higher in the mountainous regions compared to the other areas. Table 2 illustrates the descriptive statistics of meteorological variables considered in the research.

**Table 2:** Descriptive statistics.

Factors	Min.	Max.	Mean	Std. Dev.
Visibility (m)	0	14,484.00	8,470.18	4,100.06
Elevation (m)	0	969.30	205.65	233.77
Cloud cover (%)	0	100.00	55.29	46.66
Wind speed at 10-m (m/sec)	0	26.30	1.57	2.13
Precipitation (mm)	0	70.90	0.36	1.70
Dew point Depression (Kelvin)	0	39.00	1.49	2.06

Several sets of regression models (OLS and WLS) were developed based on the visibility and elevation range, by considering the weather stations close to the road network. The developed models are discussed next.

### 3.1 Visibility less than 15,000 m

Table 3 summarizes, both, OLS and WLS regression models for visibility less than 15,000 m. The regression models were developed by considering all the data near road links with visibility values less than 15,000 m. Also, based on the elevation ranges at which the weather station is located, the data were segregated, and regression models were developed using the backward elimination method. All the predictor variables presented in Table 3 are significant at a 95% confidence interval (p-value < 0.05). The results from the OLS models indicate that the elevation, cloud cover, and amount of precipitation are negatively associated with visibility. However, wind speed at 10-m AGL, dew point depression, and the presence of water bodies within 1.6 km are positively associated with visibility. The positive coefficient for wind speed indicates that visibility increases by ∼209 m for every 1 m/s increase in the wind speed. This could be attributed to boundary-layer mixing during higher wind speeds, resulting in reduced humidity leading to good visibility conditions. Likewise, rainfall in the past three hours, three to six hours, and twelve to twenty-four hours are positively associated with visibility less than 15,000 m when the complete dataset is considered. On the other hand, rainfall in the past six to twelve hours is negatively associated with the visibility in regression models based on an elevation between 50 m to 250 m and elevation between 250 m to 750 m. Further, the coefficient of precipitation for regression models based on an elevation less than 50 m is lower compared to the coefficient of precipitation for regression models based on an elevation of more than 750 m. Also, the coefficient of precipitation increases steadily with an increase in the elevation. In other words, the influence of precipitation on visibility is higher at a lower elevation compared with higher elevations if all the other predictors remain unchanged.

**Table 3:** OLS and WLS regression models for visibility data <15,000 m.

Variable	OLS					WLS				
	All	Elevation <50 m	Elevation 50 m to 250 m	Elevation 250 m to 750 m	Elevation >750 m	All	Elevation <50 m	Elevation 50 m to 250 m	Elevation 250 m to 750 m	Elevation >750 m
	Coef.	Coef.	Coef.	Coef.	Coef.	Coef.	Coef.	Coef.	Coef.	Coef.
Elevation	−1.09	-	−0.92	−1.48	−17.37	−1.10	-	−1.18	−1.50	−18.23
Cloud cover	−32.52	−34.09	−28.33	−35.44	−36.97	−33.01	−34.47	−28.23	−36.02	−38.64
Wind speed at 10-m	209.86	282.80	231.95	240.39	-	210.64	279.38	230.35	240.47	-
Precipitation	−161.89	−216.12	−141.82	−128.12	−105.95	−168.84	−225.91	−148.92	−133.94	−109.88
tair_dew	432.77	294.88	608.58	513.25	427.63	436.03	292.43	614.36	524.95	442.70
0am-4am	9,851.07	9,679.63	9,675.72	10,238.53	25,359.16	9,945.09	9,810.18	9,820.39	10,342.91	26,260.36
4am-8am	9,488.88	9,147.41	9,527.57	9,910.24	24,998.97	9,572.02	9,239.20	9,647.69	9,986.01	25,883.67
8am-12pm	8,980.28	8,670.09	8,983.95	9,327.96	24,636.44	9,031.51	8,742.02	9,074.99	9,378.47	25,454.09
12pm-4pm	9,271.29	9,041.36	9,134.62	9,632.86	24,716.27	9,319.43	9,119.31	9,197.60	9,705.09	25,545.07
4pm-8pm	8,762.38	8,672.40	8,174.97	9,454.76	24,695.88	8,805.10	8,761.18	8,229.73	9,519.71	25,502.93
8pm-12am	9,131.83	9,110.62	8,605.57	9,618.31	24,799.25	9,187.82	9,186.21	8,692.96	9,673.67	25,625.09
Rain0-3	779.91	933.00	399.94	914.04	776.27	779.30	933.70	370.77	902.89	841.47
Rain3-6	69.80	151.12	-	-	−198.67	55.68	150.13	-	-	−234.02
Rain6-12	-	271.61	−87.21	−349.60	-	-	259.16	−127.64	−373.40	-
Rain12-24	94.18	237.95	-	−137.93	375.40	95.76	237.83	-	−139.14	413.21
Water	259.19	367.98	206.83	-	-	269.34	369.33	205.99	-	-
No. of Observations	183,377	71,615	59,531	42,334	9,897	18,377	71,615	59,531	42,334	9,897
R-Squared	0.85	0.87	0.86	0.84	0.82	0.98	0.98	0.98	0.97	0.96
Adj. R-Squared	0.85	0.87	0.86	0.84	0.82	0.98	0.98	0.98	0.97	0.96
AIC	3,523,477	1,376,872	1,146,357	808,167	188,501	3,141,427	1,237,751	1,032,004	735,429	172,251
RMSE	3,598.30	3,619	3,670.60	3,381	3,360.00	1,269.70	1,370.10	1,406.30	1,430.10	1,454.70

Note: All the variables are significant at the 95% confidence level.

Similar trends were observed in the case of the WLS regression models. The coefficients of predictor variables in the OLS and WLS models are approximately consistent. In terms of statistical measures, both the OLS and WLS models are acceptable, but the developed WLS models have slightly higher R-squared and adjusted R-squared values. Also, AIC and RMSE are found to be lower for WLS models compared to OLS models. Similar results were observed in the case of models developed for different elevations.

### 3.2 Visibility less than 10,000 m

The developed OLS and WLS regression models for visibility less than 10,000 m are summarized in Table 4. The regression models were developed by considering all the data near road links with visibility values less than 10,000 m. The WLS regression models outperformed the OLS regression models based on the R-squared, Adjusted R-squared, AIC, and RMSE values. The elevation, cloud cover, the amount of precipitation and the presence of water bodies are negatively associated with visibility less than 10,000 m when the complete dataset is considered. However, elevation is observed to be positively associated with the visibility in regression models based on an elevation between 50 m to 250 m. In all the regression models, wind speed at 10-m AGL and dewpoint depression are positively associated with visibility. In addition, rainfall in the past three hours, three to six hours, six to twelve hours, and twelve to twenty-four hours is positively associated with visibility less than 10,000 m when the complete dataset is considered. However, rainfall in the past six to twelve hours and twelve to twenty-four hours is negatively associated with the visibility in regression models based on an elevation between 250 m to 750 m.

**Table 4:** OLS and WLS regression models for visibility data <10,000 m.

Variable	OLS					WLS				
	All	Elevation <50 m	Elevation 50 m to 250 m	Elevation 250 m to 750 m	Elevation >750 m	All	Elevation <50 m	Elevation 50 m to 250 m	Elevation 250 m to 750 m	Elevation >750 m
	Coef.	Coef.	Coef.	Coef.	Coef.	Coef.	Coef.	Coef.	Coef.	Coef.
Elevation	−0.49	-	1.03	−0.64	−13.87	−0.50	-	1.02	−0.68	−14.04
Cloud cover	−28.15	−28.82	−25.06	−31.11	−30.48	−28.86	−29.78	−25.61	−31.91	−30.72
Wind speed at 10-m	175.74	219.64	195.00	167.83	54.24	183.96	230.16	204.84	165.42	54.35
Precipitation	−60.44	−101.71	−41.13	−42.16	−40.13	−62.40	−101.15	−45.97	−42.21	−49.54
tair_dew	208.83	134.75	382.33	263.27	275.58	221.09	133.92	403.70	298.94	275.05
0am-4am	6,865.89	6,686.13	6,295.22	7,390.74	19,683.49	6,898.32	6,694.60	6,300.22	7,469.19	19,850.55
4am-8am	6,722.82	6,412.11	6,335.12	7,241.17	19,524.11	6,763.11	6,437.51	6,365.48	7,313.28	19,694.79
8am-12pm	6,520.76	6,294.97	6,190.45	6,829.43	19,326.89	6,542.06	6,319.06	6,201.69	6,841.30	19,504.57
12pm-4pm	6,798.39	6,634.50	6,400.06	7,118.41	19,224.01	6,824.36	6,672.49	6,422.91	7,167.27	19,373.14
4pm-8pm	6,581.29	6,411.67	5,806.57	7,273.34	19,272.53	6,590.24	6,441.66	5,804.14	7,325.95	19,422.28
8pm-12am	6,723.76	6,705.21	5,838.03	7,355.68	19,386.16	6,741.61	6,734.72	5,836.11	7,423.99	19,552.91
Rain0-3	986.64	1,130.13	657.96	1,097.47	835.14	1,000.87	1,149.07	688.43	1,108.02	859.68
Rain3-6	156.28	227.71	155.47	127.88	-	158.00	209.72	129.67	138.20	-
Rain6-12	61.84	262.39	-	−202.26	-	63.19	271.57	-	−211.21	-
Rain12-24	57.88	223.63	66.11	−181.92	216.16	66.97	218.13	58.35	−200.60	219.05
Water	−181.77	−140.86	−139.24	-	-	−195.69	−118.89	−144.38	-	-
No. of Observations	101,847	36,708	32,531	25,839	6,769	101,847	36,708	32,531	25,839	6,769
R-Squared	0.82	0.83	0.82	0.82	0.81	0.97	0.99	0.98	0.97	0.99
Adj. R-Squared	0.82	0.83	0.82	0.82	0.81	0.97	0.99	0.98	0.97	0.99
AIC	1,890,508	682,266	605,237	476,734	124,376.80	1,688,427	595,369	531,956	426,776	105,086
RMSE	2,597.00	2,628	2,652.60	2,455	2,362.20	962.97	804.63	860.03	933.68	568.16

Note: All the coefficient values presented are significant at the 95% confidence level.

### 3.3 Visibility less than 5,000 m

The developed OLS and WLS regression models for visibility less than 5,000 m are summarized in Table 5. The regression models were developed by considering all the data near road links with visibility values less than 5,000 m. In the majority of the models, elevation, cloud cover, amount of precipitation, and the presence of water bodies are negatively associated with visibility less than 5,000 m. However, elevation is positively associated with visibility less than 5,000 m in regression models based on an elevation between 50 m to 250 m. In addition, in the majority of the models, wind speed at 10-m AGL and dewpoint depression are positively associated with visibility. Also, rainfall in the past three hours, three to six hours, six to twelve hours, and twelve to twenty-four hours is positively associated with visibility less than 5,000 m when the complete dataset is considered. However, rainfall in the past six to twelve hours and twelve to twenty-four hours is negatively associated with the visibility in regression models based on an elevation between 250 m to 750 m.

**Table 5:** OLS and WLS regression models for visibility data <5,000 m.

Variable	OLS					WLS				
	All	Elevation <50 m	Elevation 50 m to 250 m	Elevation 250 m to 750 m	Elevation >750 m	All	Elevation <50 m	Elevation 50 m to 250 m	Elevation 250 m to 750 m	Elevation >750 m
	Coef.	Coef.	Coef.	Coef.	Coef.	Coef.	Coef.	Coef.	Coef.	Coef.
Elevation	-	−1.54	3.50	−0.35	−11.92	-	−1.64	3.73	−0.44	−12.61
Cloud cover	−18.14	−16.55	−16.33	−24.37	−20.57	−19.36	−17.82	−17.37	−25.36	−21.62
Wind speed at 10-m	118.75	107.30	163.22	114.42	68.10	123.84	116.01	169.63	116.03	70.90
Precipitation	-	−10.72	-	-	-	-	−11.47	-	-	-
tair_dew	15.25	17.06	37.19	-	134.33	14.96	14.01	42.79	-	127.71
0am-4am	3,884.68	3,683.15	3,013.27	4,836.86	15,265.24	3,954.84	3,762.29	3,042.41	4,925.00	15,975.83
4am-8am	3,757.55	3,480.71	2,989.43	4,638.25	15,298.57	3,827.46	3,526.27	3,031.71	4,732.54	16,006.92
8am-12pm	3,690.34	3,535.34	2,986.91	4,352.19	15,226.36	3,755.81	3,595.82	3,006.90	4,410.19	15,944.89
12pm-4pm	3,980.76	3,917.06	3,255.69	4,602.52	15,280.81	4,069.04	4,009.87	3,299.47	4,687.41	15,982.99
4pm-8pm	4,141.13	4,031.86	3,237.23	5,031.24	15,370.37	4,227.37	4,124.37	3,278.01	5,137.49	16,081.01
T8pm-12am	4,075.38	4,090.94	3,061.52	5,001.94	15,258.44	4,167.70	4,160.07	3,103.45	5,111.04	15,972.71
Rain0-3	718.88	703.84	493.85	933.22	724.73	764.13	756.75	525.06	1,003.59	742.26
Rain3-6	204.57	249.44	198.09	177.10	-	198.27	258.78	198.58	171.13	-
Rain6-12	90.56	217.31	124.58	−92.42	-	109.46	221.70	113.89	−89.43	-
Rain12-24	-	66.62	-	−106.91	-	-	64.56	-	−115.57	-
Water	−382.00	−369.09	−177.14	-	-	−413.85	−392.74	−201.22	-	-
No. of Observations	47,849	16,028	14,963	12,964	3,894	47,849	16,028	14,963	12,964	3,894
R-Squared	0.79	0.79	0.80	0.80	0.79	0.96	0.98	0.97	0.98	0.95
Adj. R-Squared	0.79	0.79	0.80	0.80	0.79	0.96	0.98	0.97	0.98	0.95
AIC	834,011	279,060	260,837	225,244	67,610	748,164	243,483	228,051	195,046	61,166
RMSE	1,474.30	1,460	1,475.40	1,434	1,423.60	601.18	481.12	493.29	447.26	622

Note: All the variables are significant at the 95% confidence level.

### 3.4 Visibility less than 2,000 m

The developed OLS and WLS regression models for visibility less than 2,000 m are summarized in Table 6. The regression models were developed by considering all the data near road links with visibility values less than 2,000 m. In the majority of the models, elevation and cloud cover are negatively associated with visibility. However, elevation is positively associated with visibility in regression models based on an elevation between 50 m to 250 m. In addition, in the majority of the models, wind speed at 10-m AGL, amount of precipitation, and dewpoint depression are positively associated with the visibility. However, dewpoint depression is negatively associated, while the presence of water bodies is positively associated with the visibility in regression models based on an elevation between 50 m to 250 m. Also, rainfall in the past three hours, three to six hours and six to twelve hours is positively associated with visibility less than 2,000 m when the complete dataset is considered. In regression models, when the complete dataset is considered, the chances of low visibility events are observed to be higher between six to twelve hours after the rainfall when compared to the first six hours after the rainfall, if all other predictor variables are kept constant. Similarly, in regression models based on an elevation less than 50 m, the chances of low visibility events are observed to be higher during the twelve to twenty-four hours after the rainfall when compared to the first three hours after the rainfall, if all other predictor variables are kept constant.

**Table 6:** OLS and WLS regression models for visibility data <2,000 m.

Variable	OLS					WLS				
	All	Elevation <50 m	Elevation 50 m to 250 m	Elevation 250 m to 750 m	Elevation >750 m	All	Elevation <50 m	Elevation 50 m to 250 m	Elevation 250 m to 750 m	Elevation >750 m
	Coef.	Coef.	Coef.	Coef.	Coef.	Coef.	Coef.	Coef.	Coef.	Coef.
Elevation	−0.03	−0.96	1.54	-	−3.75	−0.03	−1.05	1.71	-	−4.55
Cloud cover	−2.21	−2.28	−2.07	−6.09	−33.41	−2.14	−2.10	−2.36	−7.24	−35.37
Wind speed at 10-m	32.93	26.21	33.98	32.18	44.38	34.57	25.65	37.12	36.62	43.43
Precipitation	10.19	8.00	-	20.28	28.00	11.09	9.62	-	20.80	24.94
tair_dew	-	21.94	−27.37	-	85.90	-	22.87	−22.06	-	96.73
0am-4am	1,032.90	1,040.41	791.83	1,425.15	7,462.57	1,022.58	1,021.27	780.40	1,523.79	8,377.35
4am-8am	1,003.15	1,013.48	741.41	1,372.85	7,572.18	994.35	993.84	715.10	1,480.63	8,494.15
8am-12pm	902.55	935.58	657.76	1,235.84	7,495.51	887.23	916.62	635.89	1,325.42	8,422.79
12pm-4pm	863.87	861.75	653.28	1,198.26	7,461.19	838.68	832.01	631.07	1,274.96	8,388.28
4pm-8pm	1,013.51	1,025.61	757.16	1,442.44	7,525.68	1,012.60	1,010.51	728.70	1,531.63	8,440.80
8pm-12am	1,016.46	1,132.93	731.93	1,474.37	7,377.35	1,004.58	1,112.75	703.43	1,581.43	8,319.78
Rain0-3	145.06	102.49	131.49	169.85	272.25	144.49	100.23	143.49	192.33	330.23
Rain3-6	51.69	86.38	-	76.97	-	47.20	87.54	-	74.69	-
Rain6-12	22.05	-	-	-	-	22.84	-	-	-	-
Rain12-24	-	39.43	-	-	-	-	39.54	-	-	-
Water	-	-	47.03	-	-	-	-	45.50	-	-
No. of Observations	16,508	5,818	4,760	4,619	1,311	16,508	5,818	4,760	4,619	1,311
R-Squared	0.74	0.77	0.75	0.72	0.71	0.97	0.97	0.95	0.92	0.94
Adj. R-Squared	0.74	0.77	0.75	0.72	0.71	0.97	0.97	0.95	0.92	0.93
AIC	250,903	87,763	72,320	70,124	20,185	213,342	74,906	63,663	63,806	18,187
RMSE	483.04	456	481.30	479	531.13	154.85	151.00	193.86	241.49	248

Note: All the variables are significant at the 95% confidence level.

Comparing the high visibility (less than15,000 m) with low visibility (less than 2,000 m) regression models, the amount of precipitation is negatively associated with visibility less than 15,000 m, while the amount of precipitation is positively associated with visibility less than 2,000 m. In other words, the chance of visibility less than 2,000 m due to fog is lower in the event of heavy rainfall compared to a light rainfall, if all the other factors are kept constant.

Considering all the models, the WLS regression model for visibility less than 2,000 m that considers all the samples is observed to best fit the data with higher R-square and adjusted R-square values, lower AIC, and lower RMSE values. In addition, this regression model would help to understand the effect of meteorological predictor variables on low visibility conditions at link-level. It is mathematically represented as Eq. (2).

(2) Visibility = − 0.03 × (Elevation) − 2.14× (Cloud cover) + 34.57 × (Wind speed at 10-m) + 11.09 × (Precipitation) + 1,022.58 × (0am-4am) + 994.35 × (4am-8am) + 887.23 × (8am-12pm) + 838.68 × (12pm-4pm) +1,012.60 × (4pm-8pm) +1,004.58 × (8pm-12am) + 144.49 × (Rain0-3) + 47.20 × (Rain3-6) + 22.84 × (Rain6-12)

### 3.5 Comparison of visibility from different data sources

The hourly visibility values from the visibility sensor were compared with the nearest HRRR grid point, the WLS regression model based on visibility less than 2,000 m, and the nearest weather station data. In approximately four months of data collection, 25 hourly events were recorded with visibility less than 2,000 m. The selected hourly visibility values were classified as per the international standards and errors were computed by computing the difference between the visibility sensor data and respective data source as represented in Eq. (1).

**Fig. 3:**
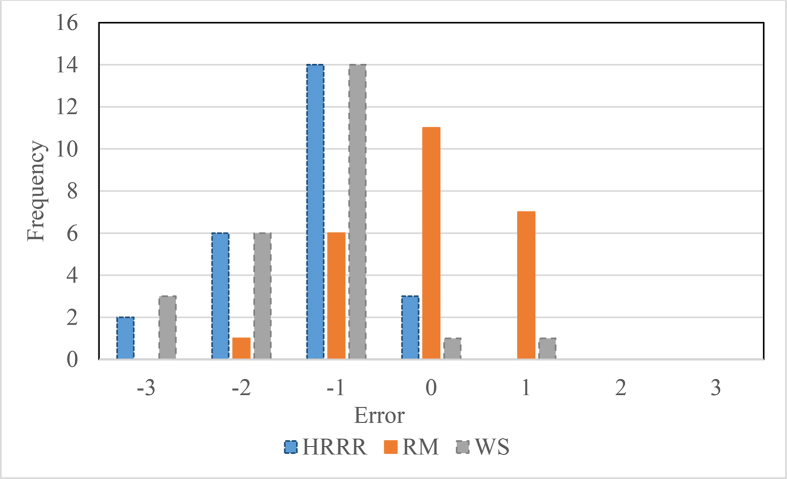
Comparison of visibility values from visibility sensor with the nearest HRRR, regression model (RM), and nearest weather station (WS).

The error histogram between the visibility classification from HRRR, regression model and weather station compared with the visibility sensor is shown in Fig. 3. The positive error indicates that the visibility at the visibility sensor is higher compared to the visibility from HRRR, regression model, and weather station, and, vice versa for the negative error. Moreover, zero error indicates that at that hour, the visibility from the sensor and HRRR, regression model, or weather station fall in the same visibility category.

The results indicate that the number of samples with zero error range was higher for the selected regression model compared to HRRR and the weather station observations. Also, the majority of the samples for HRRR and the weather station have an error of -1 (the difference between the visibility values from the visibility sensor and the respective dataset after the classification of visibility values by international standards). This indicates that the HRRR model predicts visibility one classification higher than the visibility sensor. This could be due to the distance between the HRRR grid point and the visibility sensor. The same reason applies to the error histogram associated with the weather stations data. For some locations, the nearest weather station is located at >21 km away from the visibility sensor. However, the HRRR model directly considers the real-time data from radar data and can provide two-hour forecasted weather information in real time. Therefore, this could be a possible data source for practitioners to disseminate information to motorists.

## 4 Conclusions

This paper focuses on investigating the influence and identifying factors associated with different visibility and elevation ranges at link-level. The descriptive analysis showed that the frequency of low visibility events is higher in the mountainous region when compared to other parts of NC. The WLS regression model for visibility less than 2,000 m, by considering all the samples irrespective of elevation, was considered as the best-fitted model to predict low-visibility events. In general, elevation and cloud cover are negatively associated with visibility less than 2,000 m. In addition, the chances of low visibility events are higher between six to twelve hours after rainfall when compared to the first six hours after rainfall.

The predicted air temperature, wind speed, precipitation, dew point temperature, and cloud cover are required for predicting the visibility less than 2,000 m using the developed regression model. These data elements must be captured for application purposes.

The HRRR model is a numerical weather prediction model that produces new hourly forecasts every hour and could be a solution for informing motorits of low visibility conditions. The HRRR model can help to predict visibility dynamically for the next two hours. Also, it can provide visibility information for future timestamps at a link-level. This resource could be very useful for practitioners to provide dynamic forecasts of visibility information to motorists through radio/mobile application-/dynamic signs.

In this research, most of the nearest HRRR grid points and the nearest weather stations were located within a range of 0.5 km to ∼2.0 km from the visibility sensor. Since fog is a highly localized phenomenon, the distance between each HRRR grid point, visibility sensor, and weather station would definitely play an important role when comparing visibility values from different data sources. Therefore, other approaches such as interpolating the visibility values obtained from the HRRR model and then comparing these with a significantly larger number of visibility values from the installed sensor could be considered as a possible improvement and explored in the future.

This research study focuses only on visibility related to fog events, but not reductions to visibility during heavy rainfall, dust, smoke, and wind-blown snow events. Integrating all of the aforementioned contributing factors to model low visibility under adverse weather conditions also merits an investigation.

## Declarations

### Author contribution statement

Srinivas Pulugurtha: Conceived and designed the experiments; Analyzed and interpreted the data; Wrote the paper.

Ajinkya Mane: Performed the experiments; Analyzed and interpreted the data; Wrote the paper.

Venkata Ramana Duddu: Analyzed and interpreted the data; Wrote the paper.

Christopher Godfrey: Analyzed and interpreted the data; Contributed reagents, materials, analysis tools or data; Wrote the paper.

### Funding statement

This work was supported by the North Carolina Department of Transportation (NCDOT).

### Competing interest statement

The authors declare no conflict of interest.

### Additional information

No additional information is available for this paper.
